# The maternal lifestyle in pregnancy: Implications for foetal skeletal muscle development

**DOI:** 10.1002/jcsm.13556

**Published:** 2024-08-18

**Authors:** Haijun Sun, Meixia Chen, Jialong Liao, Linjuan He, Boyang Wan, Jingdong Yin, Xin Zhang

**Affiliations:** ^1^ State Key Laboratory of Animal Nutrition and Feeding, College of Animal Science and Technology China Agricultural University Beijing China; ^2^ Institute of Animal Husbandry and Veterinary Medicine Beijing Academy of Agriculture and Forestry Sciences Beijing China; ^3^ Frontiers Science Center for Molecular Design Breeding (MOE) Beijing China

**Keywords:** foetal skeletal muscle development, gut microbiota, maternal exercise, maternal nutrition, metabolic disorders

## Abstract

The world is facing a global nutrition crisis, as evidenced by the rising incidence of metabolic disorders such as obesity, insulin resistance and chronic inflammation. Skeletal muscle is the largest tissue in humans and plays an important role in movement and host metabolism. Muscle fibre formation occurs mainly during the embryonic stage. Therefore, maternal lifestyle, especially nutrition and exercise during pregnancy, has a critical influence on foetal skeletal muscle development and the subsequent metabolic health of the offspring. In this review, the influence of maternal obesity, malnutrition and micronutrient intake on foetal skeletal muscle development is systematically summarized. We also aim to describe how maternal exercise shapes foetal muscle development and metabolic health in the offspring. The role of maternal gut microbiota and its metabolites on foetal muscle development is further discussed, although this field is still in its ‘infancy’. This review will provide new insights to reduce the global crisis of metabolic disorders and highlight current gaps to promote further research.

## Introduction

Skeletal muscle, constituting 30–40% of the body weight of a healthy adult, is the largest tissue in humans. It is heterogeneous and composed of fast‐ and slow‐twitch fibres, which differ notably in size, colour, fatigue susceptibility, oxidative metabolism capacity and mitochondrial content.^1^ Apart from its locomotive function, skeletal muscle also plays a pivotal role in glucose homeostasis. As it is the primary insulin‐responsive tissue of the body, about one third of postprandial glucose is taken up in skeletal muscle. Indeed, type 2 diabetes is characterized by insulin resistance in muscles. Conversely, targeted genetic interventions in skeletal muscle can improve metabolic homeostasis in obese mice, such as by enhancing insulin sensitivity, reducing fat deposition and alleviating non‐alcoholic fatty liver disease.^2^, ^3^ Additionally, skeletal muscle serves as a source of myokines, influencing the function of various organs, including adipose tissue, liver, pancreas and intestine, through autocrine and paracrine mechanisms.^4^, ^5^ Notably, muscle fibre formation occurs predominantly during the embryonic stage, and postnatal muscle growth primarily relies on fibre hypertrophy. This underscores the significant role of foetal skeletal muscle development in postnatal muscle growth and function.

Fat‐free mass comprises 86% of a newborn's birth weight and accounts for 83% of birth weight variation.^6^ Impaired skeletal muscle growth is a typical characteristic of foetuses with intrauterine growth restriction, ranking globally as the second leading cause of perinatal foetal mortality after premature birth.^7^ Furthermore, defects in foetal skeletal muscle development, including diminished muscle fibre quantity and area, altered muscle fibre type composition, reduced protein synthesis, decreased mitochondrial quantity and excessive triglyceride accumulation, heighten the risk of metabolic disorders in adulthood and contribute to a deterioration in life quality.^8^

Maternal lifestyle during pregnancy, particularly nutrition and physical activity, has a critical influence on the development of foetal skeletal muscle. Globally, more than 25% of women during reproductive age are overweight or obese.^9^, ^10^, ^11^ Maternal obesity (MO) can lead to a uterine environment characterized by hypoxia and inflammation, which can impede the formation of foetal muscle tissue.^12^ Conversely, maternal exercise mitigates the metabolic disturbances in offspring caused by MO.^13^ Similar to MO, the adverse effects of maternal malnutrition on foetal muscle development have been observed in rodents and livestock. Some meta‐analyses have also been conducted to evaluate micronutrient intake on pregnancy outcomes.^14^, ^15^, ^16^ Notably, there is a lack of comprehensive discussion regarding the impact of maternal nutrition and exercise on foetal skeletal muscle development. Therefore, this review aimed to investigate the effects of maternal nutrition and exercise during pregnancy on foetal skeletal muscle development. Many physiological changes in pregnant women, such as oxidative stress, low‐grade inflammation and insulin resistance, are also reflected in changes to the gut microbiota.^17^, ^18^ Therefore, this review further explored the potential role of the gut microbiota in mediating the effects of maternal lifestyle on foetal muscle development.

## Foetal skeletal muscle development

As extensively outlined in previous studies,^19^, ^20^ the myogenic progenitors of most vertebrates originate from the paraxial mesoderm and progress through transient embryonic structures (somites and myotomes) to form the extensive skeletal muscle system throughout the body. Foetal skeletal muscle formation involves complex biological processes, including the proliferation of mesodermal stem cells, determination of skeletal muscle stem cells, differentiation of myoblasts and subsequent fusion of myocytes to form multinucleated myotubes and mature muscle fibres, ultimately leading to the formation of functional muscle tissue. This developmental process is divided into primary and secondary stages. Primary myogenesis begins in the first trimester of gestation, giving rise to primary muscle fibres. Conversely, secondary myogenesis occurs in the final trimester and leads to the formation of secondary muscle fibres, which make up the main bulk of muscle tissue. Skeletal muscle formation is orchestrated by several signalling pathways, including myogenic regulatory factors (including myogenic differentiation 1 [MyoD1], myogenic factor 5 [Myf5], myogenic factor 6 [MRF4] and myogenin) and Wnt, fibroblast growth factor (FGF) and Notch signalling. A recent study has revealed that the extracellular matrix protein ADAMTS‐like 2 (ADAMTSL2) could bind to Wnt ligands and low‐density lipoprotein receptor‐related protein 6 (LRP6), thereby enhancing Wnt signalling and augmenting the expression of MyoD1, consequently promoting myoblast differentiation.^21^

A human pregnancy lasts approximately 40 weeks. Primary muscle fibres emerge at approximately the eighth week, while secondary muscle fibre formation begins after the 10th week and becomes the predominant muscle fibres by the 21st week.^22^ In mice, primary myogenesis occurs between Embryonic Days 10.5 (E10.5) and 12.5, followed by secondary myogenesis between E14.5 and E17.5.^19^ In pigs, gestation lasts approximately 114 days, with primary myogenesis from E25 to E50 and secondary myogenesis from E50 to E90.^23^ In cattle, gestation lasts ~284 days. Primary and secondary muscle fibres appear at E30 and E110, respectively. The number of total fibres is stabilized around E180.^24^ In current studies, single‐layer cells or animal models are mainly used to investigate the molecular events during myogenesis. However, multiple fundamental questions remain unaddressed due to species differences and the inability of cell cultures to fully replicate in vivo conditions. Therefore, skeletal muscle organoids cultured in three‐dimensional (3D) systems have emerged as a promising avenue for skeletal muscle development.^25^ For example, Shin and coworkers induced human pluripotent stem cells to differentiate into paraxial mesoderm using Wnt activators, bone morphogenetic protein (BMP) inhibitors and FGFs, leading to the formation of skeletal muscle organoids in a 3D culture set‐up. Encouragingly, mature muscle fibres and quiescent satellite cells were observed after prolonged cultivation of the organoids.^26^ Therefore, skeletal muscle organoids may provide a valuable platform in vitro for investigating skeletal muscle development, regeneration and the onset of muscle disorders.

## Impact of maternal nutrition on foetal skeletal muscle development

### Maternal obesity

**Figure 1:**
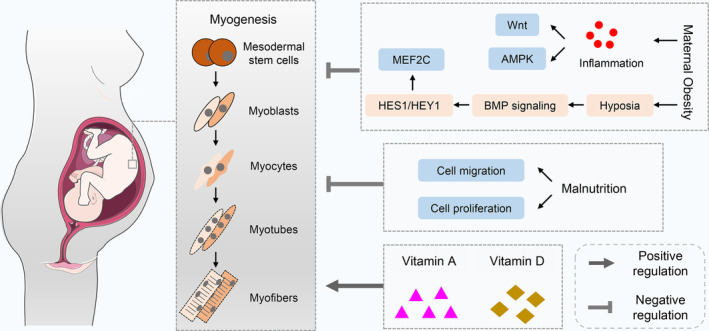
Maternal nutrition regulates foetal skeletal muscle development. Maternal obesity and malnutrition suppress the process of foetal myogenesis. Mechanically, maternal obesity induces systemic inflammation and hypoxia, leading to the inhibition of Wnt and AMPK signalling and the activation of BMP signalling. Malnutrition inhibits cell migration and proliferation. Conversely, maternal intake of vitamins A and D promotes foetal skeletal muscle development. AMPK, AMP‐activated protein kinase; BMP, bone morphogenetic protein; HES1, hes family bHLH transcription factor 1; HEY1, hes‐related family bHLH transcription factor with YRPW motif 1; MEF2C, myocyte enhancer factor 2C.

Compared to the development of the nervous system, visceral organs and bones, the development of skeletal muscle receives a lower priority in nutrient allocation, making it susceptible to nutritional fluctuations.^27^ Maternal nutrition regulates foetal skeletal muscle development mainly through influencing the fate of myogenic progenitors (*Figure* *1*). Previous reviews have explored the relationship between MO, inflammation and foetal skeletal muscle development. MO triggers low‐grade inflammation and disrupts the balance between myogenic and adipogenic differentiation of mesenchymal stem cells. This disruption occurs through the inhibition of Wnt signalling and AMP‐activated protein kinase (AMPK) signalling.^28^ In addition, 8‐week‐old female mice were fed a high‐fat diet (HFD; 45% energy from fat) for 10 weeks before mating to induce MO, and single‐cell transcriptomic analysis was used to elucidate the impact of MO on foetal skeletal muscle development in E9.5 mouse embryos. In summary, MO induced systemic embryonic hypoxia, resulting in oxidative stress, gene silencing and chromatin remodelling. Notably, MO upregulated the expression of hypoxia‐inducible factor 1 subunit alpha (HIF1A), hes family bHLH transcription factor 1 (HES1) and hes‐related family bHLH transcription factor with YRPW motif 1 (HEY1), resulting in a 63% reduction in the myogenic regulatory factor myocyte enhancer factor 2C (MEF2C).^29^ Additionally, skeletal muscle regeneration was also compromised in the offspring of obese mice. Seven days after intramuscular injection of BaCl_2_ (50 μL, 1.2% in sterile water), activation and proliferation of satellite cells in the skeletal muscle were significantly inhibited, along with a 75%, 75% and 50% reduction in MyoD, myogenin and MRF4, respectively.^30^ The m6A RNA methylation of skeletal muscle in offspring was also influenced by maternal HFD, but it remains unclear whether it is involved in cell fate switching.^31^ MO also exerts persistent adverse effects on skeletal muscle metabolism in offspring. A study in non‐human primates (Japanese macaques) has revealed that consumption of Western diets (36.6% of energy from fat and 19% from sugar) during pregnancy reduced glucose uptake capacity and suppressed the insulin receptor substrate 1 (IRS1)/protein kinase B (AKT)/TBC1 domain family member 4 (AS160) signalling pathway in foetal (E130) and neonatal (14 months old) muscle.^32^ The IRS1/AKT pathway is a central signalling pathway through which insulin plays its fundamental role in maintaining glucose homeostasis in the body.^33^ The detrimental effects of MO on skeletal muscle metabolism in the offspring of baboons,^34^ Japanese macaques^35^, ^36^ and mice^37^ have been extensively studied. It is beyond the scope of this paper and will not be discussed in detail here.

### Maternal malnutrition

Malnutrition, stemming from either inadequate food intake or illness, presents a critical health concern. It can result from an overall nutrient deficiency, energy restriction (ER) and protein limitation. Similar to MO, maternal malnutrition also impacts the proliferation of myogenic progenitors, leading to a reduction in muscle fibres. In cattle, pigs and rodents, maternal malnutrition models have been established by reducing food intake and the consumption of energy and protein. Muscle weight, oxidative capacity and insulin sensitivity in the offspring were primarily affected. An in‐depth review of this topic has been provided by Sandoval et al.^8^ A 40% food restriction in sheep during pregnancy reduced the number of myogenic progenitors in the *semitendinosus* and *triceps brachii* at different stages of embryonic development (30–65% loss), resulting in alterations in the cell cycle and gene levels related to protein synthesis.^38^ Further research demonstrated that maternal food restriction (60% of control from E45 to E100) enhanced fatty acid oxidation, reduced protein synthesis and disrupted circadian rhythms in both foetal (E100) and neonatal lambs (at 90 days old) by inhibiting the protein kinase A (PKA)–cAMP‐responsive element‐binding proteins (CREB) pathway.^39^

During pregnancy, ER in sows (reduced by 11.8%, 3.0 kcal/kg diet) resulted in a ~50% decrease in the density of primary muscle fibres at E55, thereby delaying muscle formation. In support, transcriptome sequencing analysis revealed that the expression levels of genes involved in cell migration, proliferation and organ development were reduced in the foetal muscle at E35, while the expression levels of genes negatively regulating muscle formation were increased, including myostatin and histone deacetylase 4.^40^ Research in Meishan pigs, a native pig breed in China, reported that a 12.86% reduction in dietary energy levels resulted in a reduction in foetal weight, muscle fibre density and umbilical cord blood triiodothyronine (T3) content at both E55 and E90. Aerobic metabolism in foetal skeletal muscle was also enhanced at E90, as evidenced by the increased activity of isocitrate dehydrogenase (~19%) and the decreased protein level of fast myosin heavy chain (MYH; ~35%).^41^ Conversely, in sows, elevating dietary energy levels (10.3%) before E90 also delayed foetal muscle differentiation and maturation by decreasing the expression levels of muscle growth‐related genes, MYH genes, insulin‐like growth factor 1 (IGF‐1) and IGF‐binding protein 5,^42^ indicating the critical role of appropriate maternal energy consumption in foetal skeletal muscle development.

Adverse effects of maternal protein restriction (PR) on foetal skeletal muscle development have been observed in both livestock and rodent models. In sheep, mRNA microarray analysis of foetal *longissimus dorsi* muscle showed that a 55–60% reduction in protein intake during pregnancy significantly decreased the expression of myogenic genes, including MyoD1, MYH1 and MYH2, while increasing the levels of immune‐related genes, including C‐X‐C motif chemokine ligand (CXCL) 9, CXCL10 and CXCL11.^43^ Similar findings in beef cattle further confirmed the long‐term detrimental effect of PR on offspring muscle fibres.^44^ In pregnant sows, dietary PR from 12.1% to 6.5% inhibited the formation of primary (−21.18%) and secondary muscle fibres (−20.30%) and also reduced the weight of subcutaneous fat, ultimately resulting in a 9.72% decrease in the offspring birth weight. Although the piglets regained their normal body weight at weaning, the defects in muscle fibre development were irreversible.^45^ Consistent with studies in pigs and sheep, reducing dietary protein from 17% to 6% in pregnant and lactating rats resulted in a 55% decrease in body weight, a 59% decrease in soleus muscle weight and a 66% decrease in muscle fibre area, and it impaired neuromuscular junction development in offspring at 21 days of age.^46^ Nevertheless, 1% taurine supplementation in the drinking water of pregnant mice reversed the weight loss caused by PR.^47^ At the molecular level, taurine normalized the expression of genes involved in oxidative phosphorylation.^47^ Alternatively, dietary supplementation of branched‐chain amino acids in Wistar pregnant rats ameliorated foetal growth restriction caused by PR through activating the mechanistic target of the rapamycin kinase (mTOR) signalling pathway,^48^ the master regulator of protein synthesis. It is worth noting that there is a lack of reference data on how maternal malnutrition affects foetal muscle development, and changes in the developmental trajectory of myogenic lineages remain unknown. Additionally, although amino acids are the basic units of dietary and muscle protein composition, there is a knowledge gap regarding the relationship between maternal amino acid intake and foetal muscle development. In this regard, animal models could be used to establish specific amino acid requirement parameters in future studies.

### Micronutrients

Studies on the influence of maternal micronutrients on foetal skeletal muscle development have primarily focused on vitamins. A meta‐analysis found that vitamin A intake during pregnancy could effectively enhance foetal development and reduce the risk of low birth weight (an overall reduction of 16%). In regions with a high prevalence of vitamin A deficiency, such as South Africa and East Asia, prenatal vitamin A supplementation was effective in reducing the risk of premature birth and low birth weight. Even at an intake level of 4000 mcg (RAe/day), no adverse effects were observed.^14^ A meta‐analysis of randomized controlled trials published between 1980 and 2014 found that increasing vitamin D intake during pregnancy could increase newborn birth weight (mean difference 108 g) without affecting the incidence of preeclampsia, gestational diabetes, preterm birth or low birth weight.^15^ Similarly, increasing vitamin D intake in pregnant sows (50 μg/kg vitamin D_3_ with an additional 50 μg/kg 25 OHD_3_) increased the number and area of muscle fibres in newborn piglets by increasing the expression of IGF‐2, MyoD1 and myogenin and decreasing the expression of MYH7 and myostatin.^49^ Conversely, vitamin D deficiency during pregnancy in rats resulted in a higher proportion of small muscle fibres in the newborns. Biological processes such as protein breakdown, cell proliferation and differentiation, muscle cell development and cellular cytoskeleton arrangement were affected in the offspring.^50^ The sustained effects of maternal vitamin D deficiency are more pronounced in male offspring. At 180 days of age, vitamin D deficiency (0 IU vitamin D_3_/kg) for 6 weeks and during pregnancy and lactation reduced the number of muscle fibres in the fast‐twitch muscles (extensor digitorum longus) of male offspring while increasing the number of satellite cells and the area of muscle fibres through the activation of the IGF‐1/AKT signalling pathway. However, there was no impact on the characteristics of slow‐twitch muscles (soleus). This suggests that maternal vitamin D intake may have a greater impact on glycolytic muscle fibres, along with compensatory effects on foetal skeletal muscle development after birth.^51^ In conclusion, maternal vitamin A and D intake has the potential to reduce the risk of intrauterine growth restriction in clinical practice.

In contrast to vitamins A and D, a meta‐analysis revealed that there was no association between maternal vitamin B_12_ levels during pregnancy and birth weight. However, vitamin B_12_ deficiency increased the risk of low birth weight by 15% and preterm birth by 21%.^16^ The reduced risk of low birth weight may be related to the increased bone weight. In a mouse genetic model of vitamin B_12_ deficiency, subcutaneous injection of 200 μg vitamin B_12_ during pregnancy increased bone weight with no impact on skeletal muscle weight or strength in offspring.^52^ Vitamin B_12_ is known to be a methyl donor, and therefore, its beneficial effects may be due to changes in the DNA methylation signatures of the foetus.^53^

## Impact of maternal exercise on foetal skeletal muscle development

**Figure 2:**
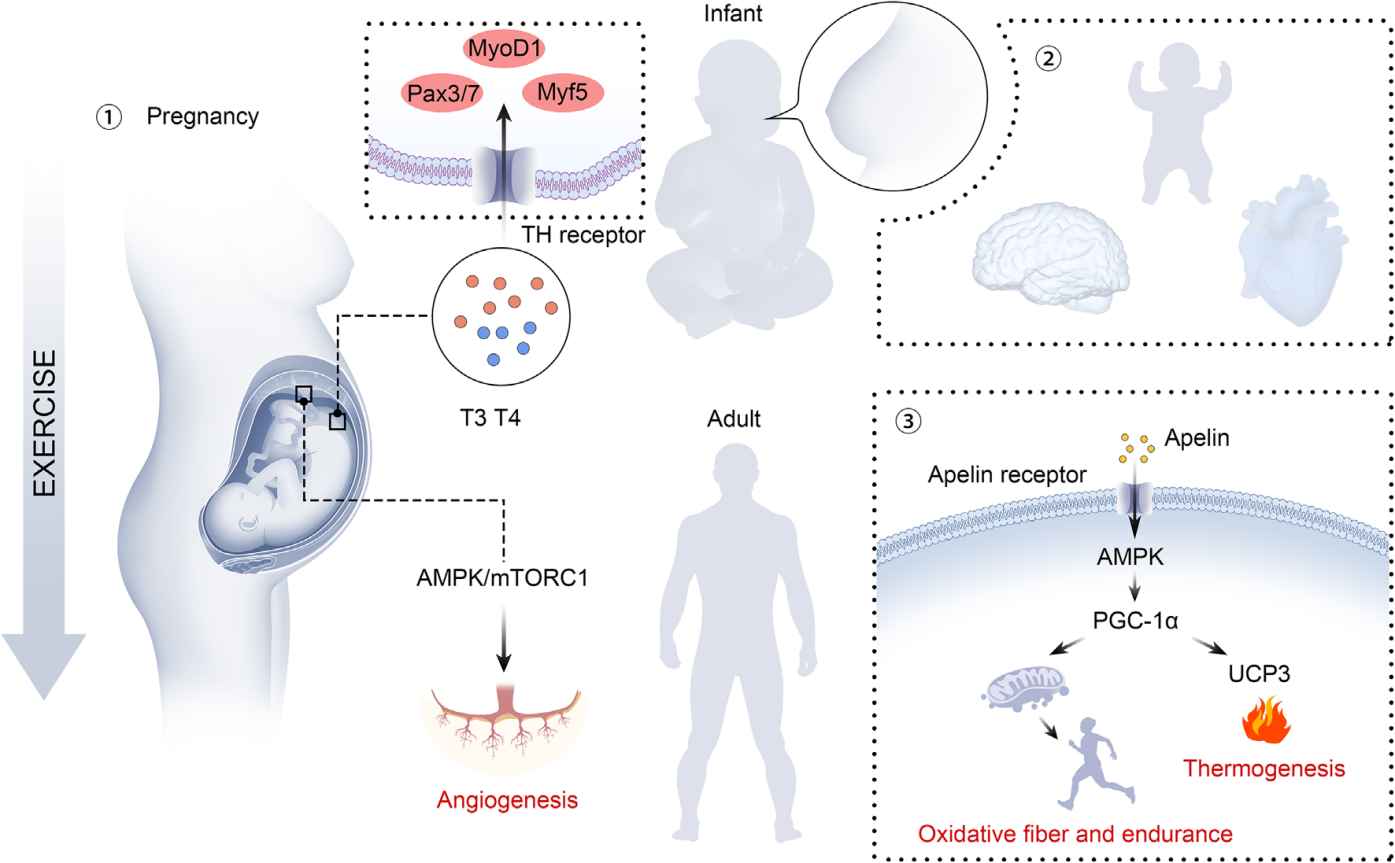
Maternal exercise contributes to foetal muscle development and metabolic health in offspring. **①** Exercise during pregnancy has a beneficial effect on placental vascular formation and foetal muscle development through thyroid hormone signalling. **②** Maternal exercise during pregnancy improves the metabolic health and cardiac function of infants through breastfeeding. **③** Maternal exercise during pregnancy elevates apelin secretion in offspring. Apelin binds with its receptor and increases AMPK activity, which enhances mitochondrial biogenesis and UCP3 expression in skeletal muscle. This biological process facilitates muscle function, including improved endurance capacity and thermogenesis. AMPK, AMP‐activated protein kinase; mTORC1, mechanistic target of rapamycin complex 1; Myf5, myogenic factor 5; MyoD1, myogenic differentiation 1; Pax, paired box; PGC‐1α, PPARG coactivator 1 alpha; T3, triiodothyronine; T4, thyroxine; TH, thyroid hormone; UCP3, uncoupling protein 3.

As extensively outlined in previous literature, both the mother and the offspring benefit from physical activities during pregnancy, particularly in preventing metabolic disorders and improving overall health.^54^, ^55^ As a result, the American College of Sports Medicine and the American College of Obstetricians and Gynecologists recommend that pregnant women participate in at least 30 min of moderate exercise daily.^56^ Maternal exercise influences both foetal skeletal muscle development and subsequent postnatal muscle function (*Figure* *2*). Embryos are unable to synthesize thyroid hormones and depend on the maternal provision of T3 and thyroxine. Maternal exercise (24 days of moderate exercise before mating and 12 days of moderate exercise during pregnancy) could induce a 1.2‐fold increase in circulating thyroid hormone levels and also facilitate their transport to the embryo. This led to the increased expression of paired box (Pax) 3/7, Myf5 and MyoD1 in the E12.5 mouse embryo, consequently resulting in a significant elevation in foetal weight. Notably, the effect of maternal exercise relied on the thyroid hormone receptor alpha (THRα), and CRISPR/Cas9‐mediated THRα knockout in the P19 embryonic cell line abolished the beneficial effect of thyroid hormones on muscle development.^57^

Moreover, wheel‐running exercise undertaken by Sprague‐Dawley rats before and throughout gestation was reported to restrain the defects of foetal muscle development induced by maternal HFD for 6 weeks before mating.^58^ The placenta serves as an interface for nutrient and oxygen exchange between mother and foetus. In obese mice, flat treadmill exercise from Days 1.5 to 16.5 of gestation, the intensity of which was set based on the maximal oxygen consumption rates, improved maternal metabolic health and enhanced placental vascularization by activating AMPK, the mechanistic target of rapamycin complex 1 (mTORC1) and insulin signalling pathways, thereby alleviating foetal insulin resistance.^59^ Interestingly, the influence of maternal exercise on offspring placental function appears to be sex specific. Microarray analysis of the mouse placenta at E18.5 revealed that 10 weeks of wheeling exercise before mating until late pregnancy suppressed lipid and steroid metabolism in the male placenta while stimulating biological processes related to muscle growth, vascular development and growth factors in the female placenta.^60^

Different factors, such as peptides and cytokines released during exercise, play a pivotal role in the offspring muscle function enhanced by maternal exercise.^61^ Apelin, a small peptide present in a variety of tissues and also produced and secreted by adipocytes, has emerged as a powerful player with potent functions in inflammation, cardiovascular disease and cancer.^62^, ^63^, ^64^ In a study published in 2020, maternal exercise was revealed to stimulate apelin production in offspring muscles. Apelin in turn enhanced muscle mitochondrial biogenesis and increased the proportion of oxidative muscle fibres and endurance, mainly by enhancing DNA demethylation in the promoter of PPARG coactivator 1 alpha (PGC‐1α) and persistently increasing its expression.^65^ However, it remains unclear whether maternal exercise directly boosts foetal muscle development through apelin. Similarly, in Wistar rats, maternal swimming (with a 2% body weight load attached to the tail) before and during pregnancy improved mitochondrial function in the skeletal muscle of adult male offspring. The impact on female offspring mainly manifested as a reduction in muscle oxidative damage,^66^ possibly due to the decreased H_2_O_2_ release from mitochondria.^67^ Non‐shivering thermogenesis in skeletal muscle and brown adipose tissue utilizes excess energy to generate heat, thereby preventing metabolic diseases such as obesity and insulin resistance. Intramuscular administration of apelin (0.5 μmol/kg/day) from E1.5 to E16.5 has also been reported to stimulate non‐shivering thermogenesis in muscle by activating AMPK signalling and uncoupling protein 3 (UCP3) expression.^68^ Additionally, maternal exercise may also contribute to the demethylation of the PR domain‐containing 16 (PRDM16) promoter through apelin, thereby increasing PRDM16 expression levels and promoting the formation of foetal brown adipose tissue. This, in turn, alleviated the metabolic disorders induced by HFD, including reducing visceral fat deposition and improving insulin sensitivity in the offspring.^13^ Therefore, the application of apelin during pregnancy may be an effective alternative to exercise for sedentary women. It should be noted that exercise intensity was generally consistent across the above‐mentioned studies. It is therefore imperative to determine whether different exercise intensities have an impact on foetal apelin production. In addition to the peptides and cytokines released during exercise, pre‐pregnancy/pregnancy exercise also improves the metabolic health of offspring through breastfeeding. In one study, C57BL/6 mice were challenged with HFD 2 weeks before mating and throughout pregnancy and divided into sedentary and exercise groups based on whether they engaged in voluntary wheel running.^69^ Results showed that offspring from sedentary mothers (housed in static cages) exhibited significantly improved metabolic health and cardiac function in adulthood after being fed milk from the exercise group, suggesting the importance of the adaptive changes in breast milk after exercise. In particular, 3′‐sialyllactose (3′‐SL) is a major component of the oligosaccharides in breast milk. Exercise within 2 months postpartum and voluntary wheel running during mouse pregnancy could both increase the content of 3′‐SL in milk. Researchers have further confirmed that 3′‐SL was a key factor in improving offspring health by using St3gal4 knockout mice (*3′‐SL* ^*−/−*^), which lack 3′‐SL in their milk. In short, milk from exercise‐trained *3′‐SL* ^*−/−*^ dams did not improve the metabolic health of wild‐type mice, including glucose intolerance, insulin sensitivity and energy expenditure.^69^

Notably, the above‐mentioned studies were primarily conducted on rodents. The influence of maternal exercise during pregnancy on foetal muscle development should be further determined through clinical trials. A meta‐analysis has demonstrated that prenatal exercise in normal‐weight women (body mass index [BMI] 18.5–24.9 kg/m^2^) could reduce the risk of preterm birth by 15% and offspring obesity by 53%. However, it had no effect on birth weight, infant weight or childhood weight for both normal‐weight and overweight women (BMI ≥ 24.9 kg/m^2^).^70^ Skeletal muscle development is precisely regulated. Embryonic samples from animal models, skeletal muscle organoids and single‐cell transcriptomics technology should be further utilized to explore the effects of maternal exercise on foetal skeletal muscle development during critical stages. Future research also needs to focus on how to translate the findings of maternal exercise to ameliorate the adverse effects of obesity from animal models to humans.

## Gut microbiota and maternal lifestyle

The gut microbiota plays a vital role in host metabolism, and its disruption could significantly impact physiology and health in humans. During pregnancy, the composition of the gut microbiota changes, particularly in the later stages of gestation. These changes include an increase in the abundance of Proteobacteria and Actinobacteria, along with a decrease in short‐chain fatty acid (SCFA) producers.^18^, ^71^, ^72^ Changes in the gut microbiota are strongly associated with weight gain, low‐grade inflammation and insulin resistance during pregnancy.^18^ Gestational diabetes, preeclampsia and preterm birth have been reported to be associated with microbial dysbiosis.^73^

In studies published in 2016 and 2020, metabolites originating from the maternal microbiota were identified in foetal faeces.^74^, ^75^ For example, higher levels of microbiota‐derived metabolites were observed in the brains, intestines and placentas of foetuses from specific pathogen‐free (SPF) mice compared to germ‐free mice. These metabolites included 5‐amino valeric acid betaine, trimethylamine oxide and tryptophan metabolites, including indole‐3‐acetic acid and indoxyl sulfate.^76^ These findings suggest that maternal microbiota and their metabolites may influence foetal development. Recently, there has been an in‐depth review regarding the influence of maternal gut microbiota on the development of the foetal immune system.^77^ While the impact of gut microbiota on skeletal muscle weight and function in adulthood has been extensively studied, research on the effects of maternal gut microbiota on foetal skeletal muscle development is relatively scarce. Nevertheless, it is evident that gut microbiota metabolites such as butyrate, lithocholic acid and indole‐3‐propionic acid significantly affect the proliferation and differentiation of myoblasts.^78^, ^79^, ^80^, ^81^ The potential effect of metabolites derived from the maternal gut microbiota on foetal skeletal muscle development warrants further investigation, as the knowledge gap is enormous.

Dietary nutrients exert a key influence on the composition and function of the gut microbiota, and this is also true for gestation. For example, a high‐fibre diet could alleviate the microbial imbalance caused by MO, thereby reducing cognitive and social dysfunction in offspring via SCFAs.^82^ Gut microbes may mediate maternal immune and metabolic responses to malnutrition,^83^ and SCFA treatment also restored the placental weight and vascularization impaired by maternal PR.^84^ Similar to MO and malnutrition, maternal vitamin intake influences the composition of the gut microbiota, particularly vitamin D. In one instance, increasing vitamin D levels during pregnancy could prevent the growth of sulfate‐reducing bacteria such as *Desulfovibrio*.^85^ Maternal vitamin D intake also has the potential to influence the infant gut microbiome, such as by increasing the abundance of *Bacteroides* and *Haemophilus*.^86^, ^87^ Furthermore, the host microbiome is also influenced by exercise. Research in rodents has shown that 8 weeks of treadmill running could increase the diversity and abundance of gut microbiota.^88^ The effects of exercise on the host gut microbiota have been extensively reviewed elsewhere.^89^, ^90^ Notably, gut microbiota diversity and gut barrier function were impaired by excessive exercise intensity and exercise in hot environments.^91^ Bridging the knowledge gap between maternal lifestyle, gut microbiota and foetal skeletal muscle development in future studies would be highly beneficial in establishing effective clinical strategies to improve muscle development.

## Conclusions

Maternal nutrition and exercise are critical for foetal skeletal muscle development. In this review, we provided a mechanistic understanding of how maternal nutrition, including obesity, malnutrition and micronutrient intake, modulates the development of foetal skeletal muscles. We also highlighted that maternal exercise shapes foetal muscle development and function through multiple pathways, including hormones, peptides or cytokines released during exercise, and breastfeeding. This field is still in its ‘infancy’, and questions for future studies include the following: Which animal and organoid models are most suitable for studying human skeletal muscle development? What are the maternal metabolic profiles during critical periods of foetal skeletal muscle development, and could dietary interventions be tailored based on this information? How about the impacts of maternal lifestyle on foetal skeletal muscle development in the face of multiple challenges such as MO and environmental exposures? For specific occupational populations exposed to di‐(2‐ethylhexyl) phthalate, bisphenol A or other environmental risk factors,^92^, ^93^, ^94^, ^95^, ^96^ is it possible to improve foetal muscle development through better nutrition and more exercise? What's more, is there a ‘healthy’ maternal microbiota for foetal skeletal muscle development, and how can we define and regulate it? In conclusion, further studies and novel technologies are needed to deepen our understanding of the interplay between maternal lifestyle, gut microbiota and foetal skeletal muscle development.

## Conflict of interest statement

The authors declare no conflicts of interest.

## References

[1] Duan Y , Li F , Tan B , Yao K , Yin Y . Metabolic control of myofibers: promising therapeutic target for obesity and type 2 diabetes. Obes Rev 2017;18:647–659. 28391659 10.1111/obr.12530

[2] Watt KI , Henstridge DC , Ziemann M , Sim CB , Montgomery MK , Samocha‐Bonet D , et al. Yap regulates skeletal muscle fatty acid oxidation and adiposity in metabolic disease. Nat Commun 2021;12:2887. 34001905 10.1038/s41467-021-23240-7 PMC8129430

[3] Guo S , Feng Y , Zhu X , Zhang X , Wang H , Wang R , et al. Metabolic crosstalk between skeletal muscle cells and liver through IRF4‐FSTL1 in nonalcoholic steatohepatitis. Nat Commun 2023;14:6047. 37770480 10.1038/s41467-023-41832-3 PMC10539336

[4] Eckel J . Myokines in metabolic homeostasis and diabetes. Diabetologia 2019;62:1523–1528. 31263909 10.1007/s00125-019-4927-9

[5] Xourafa G , Korbmacher M , Roden M . Inter‐organ crosstalk during development and progression of type 2 diabetes mellitus. Nat Rev Endocrinol 2024;20:27–49. 37845351 10.1038/s41574-023-00898-1

[6] Catalano PM , Kirwan JP . Maternal factors that determine neonatal size and body fat. Curr Diab Rep 2001;1:71–77. 12762960 10.1007/s11892-001-0013-y

[7] Năstase L , Cretoiu D , Stoicescu SM . Skeletal muscle damage in intrauterine growth restriction. Adv Exp Med Biol 2018;1088:93–106. 30390249 10.1007/978-981-13-1435-3_5

[8] Sandoval C , Wu G , Smith SB , Dunlap KA , Satterfield MC . Maternal nutrient restriction and skeletal muscle development: consequences for postnatal health. Adv Exp Med Biol 2020;1265:153–165. 32761575 10.1007/978-3-030-45328-2_9

[9] Poston L , Caleyachetty R , Cnattingius S , Corvalán C , Uauy R , Herring S , et al. Preconceptional and maternal obesity: epidemiology and health consequences. Lancet Diabetes Endocrinol 2016;4:1025–1036. 27743975 10.1016/S2213-8587(16)30217-0

[10] González‐Plaza E , Bellart J , Martínez‐Verdú MÁ , Arranz Á , Luján‐Barroso L , Seguranyes G . Pre‐pregnancy overweight and obesity prevalence and relation to maternal and perinatal outcomes. Enferm Clin (Engl Ed) 2022;32:S23–S30. 35688564 10.1016/j.enfcle.2021.04.006

[11] Kim SY , Oh SY , Sung JH , Choi SJ , Roh CR , Lee SM , et al. Validation of a strict obesity definition proposed for Asians to predict adverse pregnancy outcomes in Korean pregnant women. J Korean Med Sci 2021;36:e281. 34783214 10.3346/jkms.2021.36.e281 PMC8593408

[12] Tong JF , Yan X , Zhu MJ , Ford SP , Nathanielsz PW , Du M . Maternal obesity downregulates myogenesis and beta‐catenin signaling in fetal skeletal muscle. Am J Physiol Endocrinol Metab 2009;296:E917–E924. 19176350 10.1152/ajpendo.90924.2008 PMC2670630

[13] Son JS , Zhao L , Chen Y , Chen K , Chae SA , de Avila JM , et al. Maternal exercise via exerkine apelin enhances brown adipogenesis and prevents metabolic dysfunction in offspring mice. Sci Adv 2020;6:eaaz0359. 32494609 10.1126/sciadv.aaz0359 PMC7164955

[14] Ma G , Chen Y , Liu X , Gao Y , Deavila JM , Zhu MJ , et al. Vitamin a supplementation during pregnancy in shaping child growth outcomes: a meta‐analysis. Crit Rev Food Sci Nutr 2023;63:12240–12255. 35852163 10.1080/10408398.2022.2099810 PMC9849478

[15] Pérez‐López FR , Pasupuleti V , Mezones‐Holguin E , Benites‐Zapata VA , Thota P , Deshpande A , et al. Effect of vitamin D supplementation during pregnancy on maternal and neonatal outcomes: a systematic review and meta‐analysis of randomized controlled trials. Fertil Steril 2015;103:1278–1288. 25813278 10.1016/j.fertnstert.2015.02.019

[16] Rogne T , Tielemans MJ , Chong MF , Yajnik CS , Krishnaveni GV , Poston L , et al. Associations of maternal vitamin B12 concentration in pregnancy with the risks of preterm birth and low birth weight: a systematic review and meta‐analysis of individual participant data. Am J Epidemiol 2017;185:212–223. 28108470 10.1093/aje/kww212 PMC5390862

[17] Chen M , Zhao Y , Li S , Chang Z , Liu H , Zhang D , et al. Maternal malic acid may ameliorate oxidative stress and inflammation in sows through modulating gut microbiota and host metabolic profiles during late pregnancy. Antioxidants 2024;13:253. 38397851 10.3390/antiox13020253 PMC10886295

[18] Koren O , Goodrich JK , Cullender TC , Spor A , Laitinen K , Bäckhed HK , et al. Host remodeling of the gut microbiome and metabolic changes during pregnancy. Cell 2012;150:470–480. 22863002 10.1016/j.cell.2012.07.008 PMC3505857

[19] Chal J , Pourquié O . Making muscle: skeletal myogenesis in vivo and in vitro. Development 2017;144:2104–2122. 28634270 10.1242/dev.151035

[20] Bentzinger CF , Wang YX , Rudnicki MA . Building muscle: molecular regulation of myogenesis. Cold Spring Harb Perspect Biol 2012;4:a008342. 22300977 10.1101/cshperspect.a008342 PMC3281568

[21] Taye N , Singh M , Baldock C , Hubmacher D . Secreted ADAMTS‐like 2 promotes myoblast differentiation by potentiating WNT signaling. Matrix Biol 2023;120:24–42. 37187448 10.1016/j.matbio.2023.05.003 PMC10238107

[22] Fidziańska A . Human ontogenesis. I. Ultrastructural characteristics of developing human muscle. J Neuropathol Exp Neurol 1980;39:476–486. 7217996

[23] Ji Y , Wu Z , Dai Z , Wang X , Li J , Wang B , et al. Fetal and neonatal programming of postnatal growth and feed efficiency in swine. J Anim Sci Biotechnol 2017;8:42. 28484595 10.1186/s40104-017-0173-5 PMC5420136

[24] Bonnet M , Cassar‐Malek I , Chilliard Y , Picard B . Ontogenesis of muscle and adipose tissues and their interactions in ruminants and other species. Animal 2010;4:1093–1109. 22444612 10.1017/S1751731110000601

[25] Jalal S , Dastidar S , Tedesco FS . Advanced models of human skeletal muscle differentiation, development and disease: three‐dimensional cultures, organoids and beyond. Curr Opin Cell Biol 2021;73:92–104. 34384976 10.1016/j.ceb.2021.06.004 PMC8692266

[26] Shin MK , Bang JS , Lee JE , Tran HD , Park G , Lee DR , et al. Generation of skeletal muscle organoids from human pluripotent stem cells to model myogenesis and muscle regeneration. Int J Mol Sci 2022;23:5108. 35563499 10.3390/ijms23095108 PMC9103168

[27] Zhu MJ , Ford SP , Means WJ , Hess BW , Nathanielsz PW , Du M . Maternal nutrient restriction affects properties of skeletal muscle in offspring. J Physiol 2006;575:241–250. 16763001 10.1113/jphysiol.2006.112110 PMC1819430

[28] Du M , Yan X , Tong JF , Zhao J , Zhu MJ . Maternal obesity, inflammation, and fetal skeletal muscle development. Biol Reprod 2010;82:4–12. 19516021 10.1095/biolreprod.109.077099 PMC2802110

[29] Zhao L , Law NC , Gomez NA , Son J , Gao Y , Liu X , et al. Obesity impairs embryonic myogenesis by enhancing BMP signaling within the dermomyotome. Adv Sci (Weinh) 2021;8:e2102157. 34647690 10.1002/advs.202102157 PMC8596142

[30] Mikovic J , Brightwell C , Lindsay A , Wen Y , Kowalski G , Russell AP , et al. An obesogenic maternal environment impairs mouse growth patterns, satellite cell activation, and markers of postnatal myogenesis. Am J Physiol Endocrinol Metab 2020;319:E1008–E1018. 32954829 10.1152/ajpendo.00398.2020

[31] Li X , Yang J , Zhu Y , Liu Y , Shi X , Yang G . Mouse maternal high‐fat intake dynamically programmed mRNA m^6^A modifications in adipose and skeletal muscle tissues in offspring. Int J Mol Sci 2016;17:1336. 27548155 10.3390/ijms17081336 PMC5000733

[32] Campodonico‐Burnett W , Hetrick B , Wesolowski SR , Schenk S , Takahashi DL , Dean TA , et al. Maternal obesity and western‐style diet impair fetal and juvenile offspring skeletal muscle insulin‐stimulated glucose transport in nonhuman primates. Diabetes 2020;69:1389–1400. 32354857 10.2337/db19-1218 PMC7306120

[33] Samuel VT , Shulman GI . Mechanisms for insulin resistance: common threads and missing links. Cell 2012;148:852–871. 22385956 10.1016/j.cell.2012.02.017 PMC3294420

[34] Ampong I , Zimmerman KD , Perumalla DS , Wallis KE , Li G , Huber HF , et al. Maternal obesity alters offspring liver and skeletal muscle metabolism in early post‐puberty despite maintaining a normal post‐weaning dietary lifestyle. FASEB J 2022;36:e22644. 36415994 10.1096/fj.202201473R PMC9827852

[35] Greyslak KT , Hetrick B , Bergman BC , Dean TA , Wesolowski SR , Gannon M , et al. A maternal Western‐style diet impairs skeletal muscle lipid metabolism in adolescent Japanese macaques. Diabetes 2023;72:1766–1780. 37725952 10.2337/db23-0289 PMC10658061

[36] McCurdy CE , Schenk S , Hetrick B , Houck J , Drew BG , Kaye S , et al. Maternal obesity reduces oxidative capacity in fetal skeletal muscle of Japanese macaques. JCI Insight 2016;1:e86612. 27734025 10.1172/jci.insight.86612 PMC5053156

[37] Kelly AC , Rosario FJ , Chan J , Cox LA , Powell TL , Jansson T . Transcriptomic responses are sex‐dependent in the skeletal muscle and liver in offspring of obese mice. Am J Physiol Endocrinol Metab 2022;323:E336–E353. 35858246 10.1152/ajpendo.00263.2021 PMC9529275

[38] Gauvin MC , Pillai SM , Reed SA , Stevens JR , Hoffman ML , Jones AK , et al. Poor maternal nutrition during gestation in sheep alters prenatal muscle growth and development in offspring. J Anim Sci 2020;98:skz388. 31875422 10.1093/jas/skz388 PMC6981092

[39] Zhou X , Yan Q , Yang H , Ren A , He Z , Tan Z . Maternal intake restriction programs the energy metabolism, clock circadian regulator and mTOR signals in the skeletal muscles of goat offspring probably via the protein kinase A‐cAMP‐responsive element‐binding proteins pathway. Anim Nutr 2021;7:1303–1314. 34786503 10.1016/j.aninu.2021.09.006 PMC8567324

[40] He J , He Y , Yu B , Wang X , Chen D . Transcriptome characterization of repressed embryonic myogenesis due to maternal calorie restriction. Front Cell Dev Biol 2020;8:527. 32671071 10.3389/fcell.2020.00527 PMC7332729

[41] Zou T , He D , Yu B , Yu J , Mao X , Zheng P , et al. Moderate maternal energy restriction during gestation in pigs attenuates fetal skeletal muscle development through changing myogenic gene expression and myofiber characteristics. Reprod Sci 2017;24:156–167. 27233753 10.1177/1933719116651151

[42] Zou T , He D , Yu B , Yu J , Mao X , Zheng P , et al. Moderately increased maternal dietary energy intake delays foetal skeletal muscle differentiation and maturity in pigs. Eur J Nutr 2016;55:1777–1787. 26179476 10.1007/s00394-015-0996-9

[43] Sohel MMH , Akyuz B , Konca Y , Arslan K , Gurbulak K , Abay M , et al. Differential protein input in the maternal diet alters the skeletal muscle transcriptome in fetal sheep. Mamm Genome 2020;31:309–324. 33164111 10.1007/s00335-020-09851-3

[44] Costa TC , Du M , Nascimento KB , Galvão MC , Meneses JAM , Schultz EB , et al. Skeletal muscle development in postnatal beef cattle resulting from maternal protein restriction during mid‐gestation. Animals 2021;11:860. 33803518 10.3390/ani11030860 PMC8003034

[45] Rehfeldt C , Lefaucheur L , Block J , Stabenow B , Pfuhl R , Otten W , et al. Limited and excess protein intake of pregnant gilts differently affects body composition and cellularity of skeletal muscle and subcutaneous adipose tissue of newborn and weanling piglets. Eur J Nutr 2012;51:151–165. 21559991 10.1007/s00394-011-0201-8

[46] Confortim HD , Jerônimo LC , Centenaro LA , Pinheiro PF , Matheus SM , Torrejais MM . Maternal protein restriction during pregnancy and lactation affects the development of muscle fibers and neuromuscular junctions in rats. Muscle Nerve 2017;55:109–115. 27171684 10.1002/mus.25187

[47] Mortensen OH , Olsen HL , Frandsen L , Nielsen PE , Nielsen FC , Grunnet N , et al. A maternal low protein diet has pronounced effects on mitochondrial gene expression in offspring liver and skeletal muscle; protective effect of taurine. J Biomed Sci 2010;17:S38. 20804614 10.1186/1423-0127-17-S1-S38 PMC2994375

[48] Teodoro GF , Vianna D , Torres‐Leal FL , Pantaleão LC , Matos‐Neto EM , Donato J Jr , et al. Leucine is essential for attenuating fetal growth restriction caused by a protein‐restricted diet in rats. J Nutr 2012;142:924–930. 22457392 10.3945/jn.111.146266

[49] Zhou H , Chen Y , Lv G , Zhuo Y , Lin Y , Feng B , et al. Improving maternal vitamin D status promotes prenatal and postnatal skeletal muscle development of pig offspring. Nutrition 2016;32:1144–1152. 27209214 10.1016/j.nut.2016.03.004

[50] Max D , Brandsch C , Schumann S , Kühne H , Frommhagen M , Schutkowski A , et al. Maternal vitamin D deficiency causes smaller muscle fibers and altered transcript levels of genes involved in protein degradation, myogenesis, and cytoskeleton organization in the newborn rat. Mol Nutr Food Res 2014;58:343–352. 23963738 10.1002/mnfr.201300360

[51] Reis NG , Assis AP , Lautherbach N , Gonçalves DA , Silveira WA , Morgan HJN , et al. Maternal vitamin D deficiency affects the morphology and function of glycolytic muscle in adult offspring rats. J Cachexia Sarcopenia Muscle 2022;13:2175–2187. 35582969 10.1002/jcsm.12986 PMC9398225

[52] Singh P , Telnova S , Zhou B , Mohamed AD , Mello V , Wackerhage H , et al. Maternal vitamin B12 in mice positively regulates bone, but not muscle mass and strength in post‐weaning and mature offspring. Am J Physiol Regul Integr Comp Physiol 2021;320:R984–R993. 33759575 10.1152/ajpregu.00355.2020 PMC8285619

[53] McGee M , Bainbridge S , Fontaine‐Bisson B . A crucial role for maternal dietary methyl donor intake in epigenetic programming and fetal growth outcomes. Nutr Rev 2018;76:469–478. 29529267 10.1093/nutrit/nuy006

[54] Kusuyama J , Alves‐Wagner AB , Makarewicz NS , Goodyear LJ . Effects of maternal and paternal exercise on offspring metabolism. Nat Metab 2020;2:858–872. 32929233 10.1038/s42255-020-00274-7 PMC7643050

[55] Harris JE , Baer LA , Stanford KI . Maternal exercise improves the metabolic health of adult offspring. Trends Endocrinol Metab 2018;29:164–177. 29402734 10.1016/j.tem.2018.01.003 PMC5826804

[56] Physical activity and exercise during pregnancy and the postpartum period: ACOG Committee Opinion, Number 804. Obstet Gynecol 2020;135:e178–e188. 32217980 10.1097/AOG.0000000000003772

[57] Gao Y , Zhao L , Son JS , Liu X , Chen Y , Deavila JM , et al. Maternal exercise before and during pregnancy facilitates embryonic myogenesis by enhancing thyroid hormone signaling. Thyroid 2022;32:581–593. 35286177 10.1089/thy.2021.0639 PMC9145266

[58] Raipuria M , Bahari H , Morris MJ . Effects of maternal diet and exercise during pregnancy on glucose metabolism in skeletal muscle and fat of weanling rats. PLoS ONE 2015;10:e0120980. 25853572 10.1371/journal.pone.0120980 PMC4390148

[59] Son JS , Liu X , Tian Q , Zhao L , Chen Y , Hu Y , et al. Exercise prevents the adverse effects of maternal obesity on placental vascularization and fetal growth. J Physiol 2019;597:3333–3347. 31115053 10.1113/JP277698 PMC6602814

[60] Ruebel ML , Borengasser SJ , Zhong Y , Kang P , Faske J , Shankar K . Maternal exercise prior to and during gestation induces sex‐specific alterations in the mouse placenta. Int J Mol Sci 2023;24:16441. 38003633 10.3390/ijms242216441 PMC10671464

[61] Dubé C , Aguer C , Adamo K , Bainbridge S . A role for maternally derived myokines to optimize placental function and fetal growth across gestation. Appl Physiol Nutr Metab 2017;42:459–469. 28177716 10.1139/apnm-2016-0446

[62] Wang X , Zhang L , Li P , Zheng Y , Yang Y , Ji S . Apelin/APJ system in inflammation. Int Immunopharmacol 2022;109:108822. 35605524 10.1016/j.intimp.2022.108822

[63] Chapman FA , Maguire JJ , Newby DE , Davenport AP , Dhaun N . Targeting the apelin system for the treatment of cardiovascular diseases. Cardiovasc Res 2023;119:2683–2696. 37956047 10.1093/cvr/cvad171 PMC10757586

[64] Yang Y , Lv SY , Ye W , Zhang L . Apelin/APJ system and cancer. Clin Chim Acta 2016;457:112–116. 27083318 10.1016/j.cca.2016.04.001

[65] Son JS , Chae SA , Wang H , Chen Y , Bravo Iniguez A , de Avila JM , et al. Maternal inactivity programs skeletal muscle dysfunction in offspring mice by attenuating apelin signaling and mitochondrial biogenesis. Cell Rep 2020;33:108461. 33264618 10.1016/j.celrep.2020.108461 PMC8137280

[66] Hözer RM , Dos Santos BG , August PM , Rodrigues KS , Maurmann RM , Flores EB , et al. Maternal exercise during pregnancy modulates mitochondrial function and redox status in a sex‐dependent way in adult offspring's skeletal muscle. J Dev Orig Health Dis 2022;13:204–211. 33947489 10.1017/S2040174421000209

[67] Siti F , Dubouchaud H , Hininger I , Quiclet C , Vial G , Galinier A , et al. Maternal exercise before and during gestation modifies liver and muscle mitochondria in rat offspring. J Exp Biol 2019;222:jeb194969. 31019067 10.1242/jeb.194969

[68] Son JS , Chae SA , Zhao L , Wang H , de Avila JM , Zhu MJ , et al. Maternal exercise intergenerationally drives muscle‐based thermogenesis via activation of apelin‐AMPK signaling. EBioMedicine 2022;76:103842. 35081489 10.1016/j.ebiom.2022.103842 PMC8790600

[69] Harris JE , Pinckard KM , Wright KR , Baer LA , Arts PJ , Abay E , et al. Exercise‐induced 3′‐sialyllactose in breast milk is a critical mediator to improve metabolic health and cardiac function in mouse offspring. Nat Metab 2020;2:678–687. 32694823 10.1038/s42255-020-0223-8 PMC7438265

[70] Chen Y , Ma G , Hu Y , Yang Q , Deavila JM , Zhu MJ , et al. Effects of maternal exercise during pregnancy on perinatal growth and childhood obesity outcomes: a meta‐analysis and meta‐regression. Sports Med 2021;51:2329–2347. 34143412 10.1007/s40279-021-01499-6

[71] Calatayud M , Koren O , Collado MC . Maternal microbiome and metabolic health program microbiome development and health of the offspring. Trends Endocrinol Metab 2019;30:735–744. 31493988 10.1016/j.tem.2019.07.021

[72] Vento‐Tormo R , Efremova M , Botting RA , Turco MY , Vento‐Tormo M , Meyer KB , et al. Single‐cell reconstruction of the early maternal‐fetal interface in humans. Nature 2018;563:347–353. 30429548 10.1038/s41586-018-0698-6 PMC7612850

[73] Turjeman S , Collado MC , Koren O . The gut microbiome in pregnancy and pregnancy complications. Curr Opin Endocr Metab Res 2021;18:133–138.

[74] Gomez de Agüero M , Ganal‐Vonarburg SC , Fuhrer T , Rupp S , Uchimura Y , Li H , et al. The maternal microbiota drives early postnatal innate immune development. Science 2016;351:1296–1302. 26989247 10.1126/science.aad2571

[75] Li Y , Toothaker JM , Ben‐Simon S , Ozeri L , Schweitzer R , McCourt BT , et al. In utero human intestine harbors unique metabolome, including bacterial metabolites. JCI Insight 2020;5:e138751. 33001863 10.1172/jci.insight.138751 PMC7710283

[76] Pessa‐Morikawa T , Husso A , Kärkkäinen O , Koistinen V , Hanhineva K , Iivanainen A , et al. Maternal microbiota‐derived metabolic profile in fetal murine intestine, brain and placenta. BMC Microbiol 2022;22:46. 35130835 10.1186/s12866-022-02457-6 PMC8819883

[77] Koren O , Konnikova L , Brodin P , Mysorekar IU , Collado MC . The maternal gut microbiome in pregnancy: implications for the developing immune system. Nat Rev Gastroenterol Hepatol 2024;21:35–45. 38097774 10.1038/s41575-023-00864-2 PMC12635954

[78] Chen S , Huang L , Liu B , Duan H , Li Z , Liu Y , et al. Dynamic changes in butyrate levels regulate satellite cell homeostasis by preventing spontaneous activation during aging. Sci China Life Sci 2024;67:745–764. 38157106 10.1007/s11427-023-2400-3

[79] Ding Y , Wang P , Li C , Zhang Y , Yang C , Zhou X , et al. Sodium butyrate induces mitophagy and apoptosis of bovine skeletal muscle satellite cells through the mammalian target of rapamycin signaling pathway. Int J Mol Sci 2023;24:13474. 37686278 10.3390/ijms241713474 PMC10487490

[80] Sun L , Li F , Tan W , Zhao W , Li Y , Zhu X , et al. Lithocholic acid promotes skeletal muscle regeneration through the TGR5 receptor. Acta Biochim Biophys Sin (Shanghai) 2023;55:51–61. 36647725 10.3724/abbs.2022201 PMC10157625

[81] Du L , Qi R , Wang J , Liu Z , Wu Z . Indole‐3‐propionic acid, a functional metabolite of clostridium sporogenes, promotes muscle tissue development and reduces muscle cell inflammation. Int J Mol Sci 2021;22:12435. 34830317 10.3390/ijms222212435 PMC8619491

[82] Liu X , Li X , Xia B , Jin X , Zou Q , Zeng Z , et al. High‐fiber diet mitigates maternal obesity‐induced cognitive and social dysfunction in the offspring via gut‐brain axis. Cell Metab 2021;33:923–938. 33651981 10.1016/j.cmet.2021.02.002

[83] Connor KL , Chehoud C , Altrichter A , Chan L , DeSantis TZ , Lye SJ . Maternal metabolic, immune, and microbial systems in late pregnancy vary with malnutrition in mice. Biol Reprod 2018;98:579–592. 29324977 10.1093/biolre/ioy002

[84] Pronovost GN , Yu KB , Coley‐O'Rourke EJL , Telang SS , Chen AS , Vuong HE , et al. The maternal microbiome promotes placental development in mice. Sci Adv 2023;9:eadk1887. 37801498 10.1126/sciadv.adk1887 PMC10558122

[85] Aparicio A , Gold DR , Weiss ST , Litonjua AA , Lee‐Sarwar K , Liu YY . Association of vitamin D level and maternal gut microbiome during pregnancy: findings from a randomized controlled trial of antenatal vitamin D supplementation. Nutrients 2023;15:2059. 37432235 10.3390/nu15092059 PMC10181263

[86] Molani‐Gol R , Rafraf M . Maternal vitamin D in pregnancy and infant's gut microbiota: a systematic review. Front Pediatr 2023;11:1248517. 37915988 10.3389/fped.2023.1248517 PMC10617198

[87] Drall KM , Field CJ , Haqq AM , de Souza RJ , Tun HM , Morales‐Lizcano NP , et al. Vitamin D supplementation in pregnancy and early infancy in relation to gut microbiota composition and *C. difficile* colonization: implications for viral respiratory infections. Gut Microbes 2020;12:1799734. 32779963 10.1080/19490976.2020.1799734 PMC7524344

[88] Kim D , Kang H . Exercise training modifies gut microbiota with attenuated host responses to sepsis in wild‐type mice. FASEB J 2019;33:5772–5781. 30702933 10.1096/fj.201802481R

[89] Monda V , Villano I , Messina A , Valenzano A , Esposito T , Moscatelli F , et al. Exercise modifies the gut microbiota with positive health effects. Oxid Med Cell Longev 2017;2017:1–8. 10.1155/2017/3831972 PMC5357536 28357027

[90] Mach N , Fuster‐Botella D . Endurance exercise and gut microbiota: a review. J Sport Health Sci 2017;6:179–197. 30356594 10.1016/j.jshs.2016.05.001 PMC6188999

[91] Chantler S , Griffiths A , Matu J , Davison G , Jones B , Deighton K . The effects of exercise on indirect markers of gut damage and permeability: a systematic review and meta‐analysis. Sports Med 2021;51:113–124. 33201454 10.1007/s40279-020-01348-y PMC7806566

[92] Lee DW , Lim YH , Shin CH , Lee YA , Kim BN , Kim JI , et al. Prenatal exposure to di‐(2‐ethylhexyl) phthalate and decreased skeletal muscle mass in 6‐year‐old children: a prospective birth cohort study. Environ Res 2020;182:109020. 31863942 10.1016/j.envres.2019.109020

[93] Li F , Luo T , Rong H , Lu L , Zhang L , Zheng C , et al. Maternal rodent exposure to di‐(2‐ethylhexyl) phthalate decreases muscle mass in the offspring by increasing myostatin. J Cachexia Sarcopenia Muscle 2022;13:2740–2751. 36263449 10.1002/jcsm.13098 PMC9745490

[94] Chen G , Xiong S , Jing Q , van Gestel CAM , van Straalen NM , Roelofs D , et al. Maternal exposure to polystyrene nanoparticles retarded fetal growth and triggered metabolic disorders of placenta and fetus in mice. Sci Total Environ 2023;854:158666. 36108837 10.1016/j.scitotenv.2022.158666

[95] Alonso‐Magdalena P , Vieira E , Soriano S , Menes L , Burks D , Quesada I , et al. Bisphenol A exposure during pregnancy disrupts glucose homeostasis in mothers and adult male offspring. Environ Health Perspect 2010;118:1243–1250. 20488778 10.1289/ehp.1001993 PMC2944084

[96] García‐Arevalo M , Alonso‐Magdalena P , Rebelo Dos Santos J , Quesada I , Carneiro EM , Nadal A . Exposure to bisphenol‐A during pregnancy partially mimics the effects of a high‐fat diet altering glucose homeostasis and gene expression in adult male mice. PLoS ONE 2014;9:e100214. 24959901 10.1371/journal.pone.0100214 PMC4069068

